# Fueling Work Engagement: The Role of Sleep, Health, and Overtime

**DOI:** 10.3389/fpubh.2021.592850

**Published:** 2021-05-20

**Authors:** Ricarda Schleupner, Jana Kühnel

**Affiliations:** Occupational, Economic and Social Psychology, University of Vienna, Vienna, Austria

**Keywords:** sleep, work engagement, mental health, job demands - resources model, resources

## Abstract

With the current study, we investigate mechanisms linking sleep quality with work engagement. Work engagement is an affective-motivational state of feeling vigorous, absorbed, and dedicated while working. Drawing from both the effort-recovery model and the job demands-resources framework, we hypothesize that sleep quality should be positively related to work engagement *via* the replenishment of personal resources that become apparent in mental health and physical health. Because personal resources should gain salience especially in the face of job demands, we hypothesize that overtime as an indicator for job demands should strengthen the positive relationship between mental health and work engagement. We gathered data from 152 employees from diverse industries *via* an online survey. Results showed that sleep quality was positively related to work engagement (*r* = 0.20, *p* < 0.05), and that mental health mediated this relationship (indirect effect: β = 0.23, lower limit confidence interval = 0.13, upper limit confidence interval = 0.34). However, physical health did not serve as a mediator. Overtime turned out to be significantly and positively related to work engagement (*r* = 0.22, *p* < 0.01), replicating previous findings, but did not significantly interact with mental health or physical health in predicting work engagement. Overall, the study highlights the significance of sleep quality for employees' mental health and work engagement.

## Introduction

The research on sleep and its relationship to factors in the context of work has gained growing attention over the past years. Sleep at night has been acknowledged to play an important role for employees' experiences and behavior at work (1–4). However, little is yet known about the mechanisms linking employees' sleep with their experience and behavior at work, i.e., how sleep affects an employee's experience and behavior. Among others, energetic and self-regulatory resources (5) have been proposed as mechanisms for the relationship between sleep and work engagement. Yet, in order to draw implications for practice and to highlight sleep's significance for employees' health and performance, it is important to gain an even better understanding of the ways in which sleep can influence employees' behavior. This paper aims to contribute to existing research in several ways. First, it aims to contribute to sleep research by shedding light on possible mechanisms, namely mental health and physical health, between sleep quality and well-being at work, represented through work engagement. Second, we would like to broaden the perspective on the *Job Demands-Resources* (JD-R) model (6, 7) and examine mental and physical health as personal, non-work resources in our conceptual model that link the non-work domain, represented through sleep, and the work domain, represented by work engagement. Also, we would like to examine if indicators of job demands can have similar effects for the relationships within the JD-R as have job demands themselves. Third, we would like to point out the opportunity sleep offers as a self-regulation strategy for improving an employee's own health and performance. E.g., organizations may offer trainings or speeches on topics like sleep hygiene and recovery after work and implement work conditions which support employees in pursuing a healthy lifestyle.

**Figure 1:**
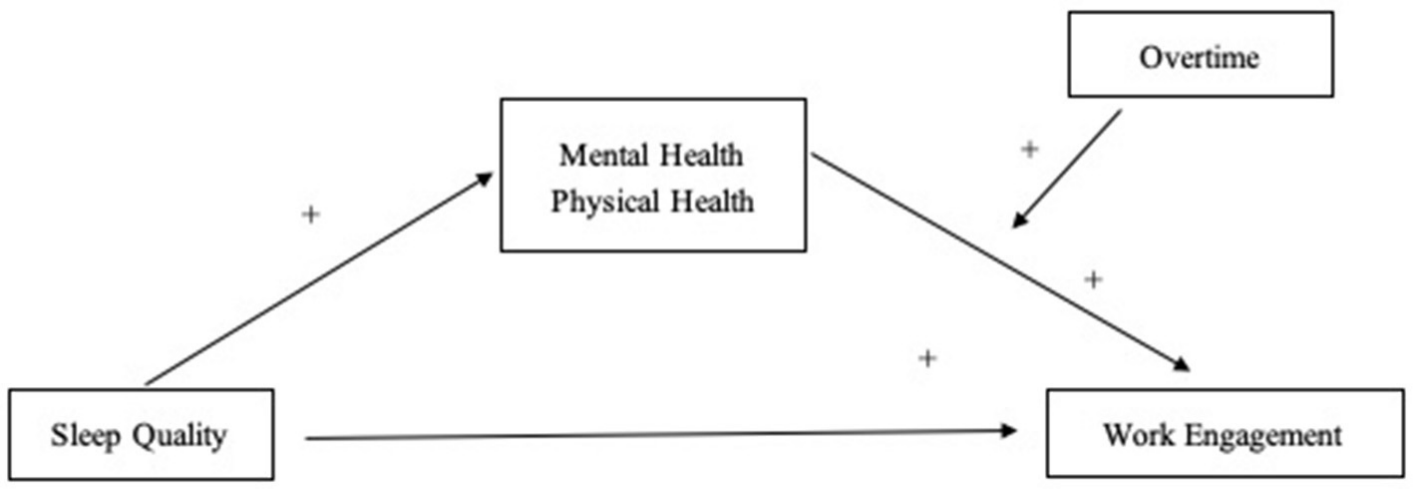
Conceptual model of this study. The “+” indicates that a positive relationship/moderating effect is assumed.

In the following sections, we derive our hypotheses on the relationships between sleep, work engagement, mental and physical health, and overtime from the Job Demands-Resources framework. Figure 1 shows the conceptual model for this study and summarizes our hypotheses. We are taking a perspective in line with the Positive Occupational Psychology framework (8, 9) focusing on positive outcomes and opportunities for promoting employees' mental and physical health and work engagement.

### Sleep at Night and Work Engagement

Everybody needs sleep (10). Especially after facing cognitive or physical demands, the human body and mind need rest to restore energy and resources, e.g., self-regulatory or affective resources, and process new information (Effort-Recovery model) (11). Several studies have shown the crucial impact sleep has on mental and physical recovery processes (12–14). Sleep deprivation, on the other hand, can lead to serious consequences for health and performance (10).

Sleep quality appears to be an important predictor for employees' behavior and experience at work, including one of the key concepts in work and health research of the past decade, which is work engagement (15). Bakker, Schaufeli and Salanova (16) define work engagement as the experience of vigor, dedication and absorption at work. Work engagement takes a central role in the JD-R (6, 7) as a desirable, positive outcome being positively associated with an employee's performance (8) contrary to depletion and burnout as negative outcomes.

According to the JD-R model, the presence and availability of resources promotes work engagement. Following the Effort-Recovery model (11) sleep is responsible for restoring resources depleted during the day. Based on this theory, we assume that sleep is positively linked to work engagement *via* the regeneration of resources. People who sleep better should have more resources available during the day. Thus, they experience more work engagement compared to people who sleep worse. Indeed, the positive relationship between sleep quality and work engagement has already been addressed and supported in previous studies (5, 17–19). Theoretical ideas like both the JD-R framework and the Effort-Recovery model, supported by empirical results, indicate that recharging resources is a linking mechanism in this relationship. Therefore, the relationship between sleep quality and work engagement is the starting point for our hypotheses.

*Hypothesis 1*. The better employees sleep, the higher is their work engagement.

### Sleep at Night, Mental Health, and Physical Health

Sleep at night is crucial for recovery, regenerating resources and thus, for mental and physical health (10). According to Meijman's and Mulder's Effort-Recovery model (11), especially after a demanding and resource-depleting workday, it is important to recover (*load effect*), i.e., to sleep well and sufficiently, in order to avoid negative *load consequences*, e.g., for employees' health. Several studies report health impairments after sleep deprivation (20). Reid et al. found sleep to be a significant predictor of both mental and physical health in a sample consisting of elderly people, such that poor sleep quality resulted in poor mental and physical health (21). A 30-day experiment on sleep quality and quantity and mental health showed similar results for young people and students (22). Insomnia and nightmares were found to serve as predictors of impaired mental health, such as paranoia, hallucinations, anxiety, depression, and mania (23). Equivalently, Freeman et al. showed how improving sleep through a Cognitive Behavioral Therapy sleep intervention including measures of sleep hygiene helped reducing symptoms of paranoia and hallucinations (24). Health practices like sufficient restorative sleep, physical exercise, and regular and healthy meals also help with improving the physical health status independently of age, sex, and economic status (25). According to a study on sleep's consequences for physical health, the functioning of T cells is regenerated during sleep, strengthening the immune system and the effectiveness of fighting pathogens (26).

Following our theoretical argumentation based on the effort-recovery model and supported by previous empirical findings, sleep quality should be positively related to both mental and physical health.

*Hypothesis 2*. The better sleep employees experience, the healthier they are (a) mentally and (b) physically.

### Health as a Personal Resource That Enables the Experience of Work Engagement

The presence and accessibility of resources plays a significant role for the extent of work engagement an employee experiences (6). Those resources can be job-related or of personal nature (15). Hobfoll defines a personal resource to be anything that helps individuals to affect and control their environment in order to meet demands in their daily life (27). However, up until now, most studies building on the JD-R framework have examined job-related resources (28). Even personal resources investigated were often indirectly linked to the job context, e.g., an employee's career progression and planning (29) or organizational-based self-esteem (30). However, personal resources play at least an equally important role within the JD-R model as do job resources (15). Xanthopoulou et al. showed that personal resources are positively linked to the perception of accessible job resources (30), implying that personal resources might be even more important. Therefore, we would like to broaden the perspective on the connections within the JD-R model and propose that health is a personal, non-work-related resource and predictor of work engagement.

Health is defined by the World Health Organization (WHO) as “a resource for everyday life, not the objective of living” and “a positive concept emphasizing social and personal resources, as well as physical capacities” (31). This definition reflects the salutogenic perspective on health that has been established over the past decades. Furthermore, it provides a first hint of health being a personal resource. Health can be further regarded as mental or physical. “Mental health includes […] emotional, psychological, and social well-being” (32). Ergo, mental health makes up a significant part of psychosocial functioning (33). Physical health means “the condition of [the] body, taking into consideration everything from the absence of disease to fitness level. [It] is critical for overall well-being […]” (34). The definitions show that mental health and physical health both contribute to the overall health state in their own way, but still, they have a (psychosomatic) connection. Therefore, it is necessary to investigate both mental health and physical health when talking about overall health. Perceived mental and physical health have been found to be related to work engagement (8, 35). Similarly, poor health states can result in reduced productivity (36), job performance, and increased sickness absence, a fact that was acknowledged by developing an instrument to calculate workplace costs caused by health problems (HPQ) (37) and by initiating the annual German Report on Absence from Work (38).

Considering an employee's mental and physical health as important personal resources to draw from, according to the JD-R model, both mental and physical health should be positively linked to work engagement.

*Hypothesis 3*. The healthier employees are (a) mentally and (b) physically, the more work engagement they show.

One of our aims in the current study is to identify mechanisms between sleep and work engagement. More specifically, we examine how or why sleep quality affects work engagement. Therefore, we hypothesize that both mental health and physical health serve as mediators between sleep quality and work engagement based on the following arguments: First, sleep is positively linked to work engagement *via* the restoration of personal resources (39). Second, sleep is both responsible for restoring personal resources and maintaining mental and physical health (Hypothesis 2), which themselves are personal resources that enable the experience of work engagement (Hypothesis 3). These considerations result in Hypothesis 4.

*Hypothesis 4*. Employees' sleep quality is indirectly and positively related to work engagement *via* (a) better mental health and (b) better physical health.

### Overtime as an Indicator for Job Demands

According to the JD-R model, another important aspect when explaining the experience of work engagement is the role of job demands. In the JD-R model, resources are assumed to increase work engagement in employees. Contrary to this, job demands are assumed to cause depletion and burnout (6).

Long work hours are an especially interesting factor to consider when investigating job demands, because they are an indicator for several possible job demands an employee might experience. E.g., high workload and time pressure might cause an employee to work overtime. Overtime hours have been used as an indicator for high workload in several empirical studies (40, 41). Overall, overtime and high workload have been found to have similar effects on employees' health (42): Like job demands, overtime hours can cause strain in employees, raise their cortisol levels and threaten their balance between work and non-work periods (40, 43, 44). We would like to test if overtime as an indicator for job demands with its own potential of damaging employees' health can equally serve as “job demand” within the JD-R model as do job demands themselves.

According to the JD-R theory's *Coping Hypothesis*, resources gain particular salience in the face of demands (7, 45). This means that job demands trigger employees to invest more resources into their work and this investment leads to even higher work engagement. We expect this to be also true for health as a personal resource and overtime as an indicator for job demands. Results of empirical studies have shown that good health conditions, such as low depression, fatigue or anxiety, good recuperation and well-being, are associated with beneficial coping behavior (46, 47). Consequently, both mental health and physical health should contribute to successfully coping with job demands, like time pressure or high workload, and therefore also with overtime hours. When confronted with job demands, health provides resources that employees can invest in the effort of coping. Hence, being confronted with job demands becoming apparent in overtime hours should cause employees to invest even more mental resources and physical energy into their work. This way, they enable themselves to deal with the demand and therefore boost the effect of good mental and physical health, resulting in even higher work engagement.

*Hypothesis 5a*. The relationship between mental health and work engagement is stronger for employees who work more overtime hours.

*Hypothesis 5b*. The relationship between physical health and work engagement is stronger for employees who work more overtime hours.

## Materials and Methods

### Sample and Procedure

One hundred and fifty-two employees (91 females, 61 males) from companies operating in diverse industries [the top three being services (*N* = 38); education (*N* = 24); public health/social affairs (*N* = 22)] participated in our study. They were recruited for a master thesis project and did not receive compensation for their participation. All subjects gave their informed consent before they participated in the study. The study was conducted in accordance with the ethical guidelines for the treatment of human subjects of the German Psychological Association (48) and the model code of ethics of the European Federation of Psychologists' Associations (49). The participants' age ranged from 19 to 69 (*M* = 37.91, *SD* = 12.35). 19.7% of the sample had children and 32.9% were in a leadership position. The sample exclusively consisted of non-shift workers. The regular weekly working hours in our sample ranged from 35 to 55 h (*M* = 39.44, *SD* = 5.47) as indicated by participants, while the actual weekly working hours ranged from 35 to 70 h (*M* = 43.71, *SD* = 6.66).

The questionnaire was provided *via* an online survey platform, so participants filled it in using electronic devices. The questionnaire consisted of scales or items retrieved from existing and reliable instruments that are described below.

### Measures

#### Sleep Quality

We assessed sleep quality using a single item (“Please evaluate your overall sleep quality during the last 4 weeks”) from the Pittsburgh Sleep Quality Index (PSQI) (50). This item has been successfully used in previous studies (5, 51) because it represents the core indicator for sleep quality (52). A single item to assess sleep quality shows high correlations with the Morning Questionnaire-Insomnia (MQI) and the PSQI overall score (53). The item-total correlation of the single item assessing sleep quality with all other components of the PSQI is high (*r* = 0.73, *p* < 0.001) (54). The rating scale for the sleep quality item ranged from 1 = *very bad* to 5 = *very good*.

#### Work Engagement

We used the 9-item version of the Utrecht Work Engagement Scale, UWES-9 (16). The instrument contains three items for each of the three subcomponents of work engagement, vigor (e.g., “At my work, I feel bursting with energy”), absorption (e.g., “I get carried away when I am working”), and dedication (e.g., “My job inspires me”). Participants answered the items using a Likert scale ranging from 0 = *never* to 6 = *always / every day*. Cronbach's alpha was 0.94.

#### Mental and Physical Health

We used the German 12-item version (55) of the Short-Form Health Survey, SF-12 (56), to assess both mental and physical health. The questionnaire consists of two subscales, one for mental and one for physical health, with six items each. An example item from the mental health subscale is “Have you felt downhearted and low?,” an example item from the physical health subscale is “The following questions are about activities you might do during a typical day. Does your health limit you in these activities? If so, how much?—Climbing several flights of stairs.” Some items should be answered with regard to the last 4 weeks, others are asked in general. Scale ranges vary between two (*yes / no*) and six points (*All of the time* to *none of the time*). A sum score is computed from all items for each of the two subscales, resulting in scores between 0 and 100, high scores representing a good health state. Cronbach's alpha was 0.73 for the physical health subscale and 0.82 for the mental health subscale.

#### Overtime

Participants indicated the number of hours they actually spent at work each week, in contrast to the number of work hours per week that was agreed on by contract. The difference between the two indicators equals the number of overtime hours.

### Statistical Analyses

To test Hypotheses 1 – 3 and 5, we conducted regression analyses using the software SPSS Statistics (IBM) to predict work engagement. For the analysis of Hypothesis 3, we simultaneously entered both mental health and physical health as predictors of work engagement into the regression model. We used the PROCESS macro for SPSS (57) to test for the mediation and moderation effects predicted in Hypotheses 4 and 5.

## Results

### Correlation Analyses

In line with our expectations, sleep quality was significantly and positively correlated with work engagement (*r* = 0.20, *p* < 0.05), mental health (*r* = 0.48, *p* < 0.001), and physical health (*r* =0.25, *p* < 0.01; see Table 1). Table 1 also shows that mental health was significantly and positively related to work engagement (*r* = 0.46, *p* < 0.01), but that physical health was not significantly related to work engagement (*r* = 0.02, *p* = 0.781). Further, overtime was significantly related to work engagement (*r* = 0.22, *p* < 0.01).

**Table 1:** Means, standard deviations, and correlations of variables.

**Variable**	***M***	***SD***	**1**	**2**	**3**	**4**	**5**	**6**	**7**	**8**	**9**
1. Sleep quality	3.43	0.96									
2. Work engagement	3.48	1.21	0.20^*^								
3. Mental health	46.14	9.86	0.48^***^	0.46^***^							
4. Physical health	52.93	7.06	0.25^**^	0.02	0.02						
5. Overtime (in hours)	5.05	9.32	0.04	0.22^**^	0.08	0.12					
6. Age	37.91	12.35	0.19^*^	0.10	0.33^***^	0.05	0.18^*^				
7. Gender	0.40	0.49	0.25^**^	−0.05	0.11	0.11	0.21^**^	0.29^***^			
8. Leadership position	0.37	0.49	0.09	0.12	0.13	0.00	0.34^***^	0.44^***^	0.30^**^		
9. Working hours (regular)	39.44	5.47	0.07	0.05	0.16^*^	0.08	0.04	0.14	0.14	0.29^**^	
10. Working hours (actual)	43.71	6.66	0.02	0.20^*^	0.11	0.13	0.87^***^	0.22^**^	0.21^**^	0.46^***^	0.52^***^

*N = 152. Gender is coded 0 = female, 1 = male. Leadership position is coded 0 = no leadership position, 1 = leadership position*.

* *p < 0.05*.

** *p < 0.01*.

*** *p < 0.001*.

### Test of Hypotheses

Results of regression analyses to test Hypotheses 1 – 3 and 5 are depicted in Table 2. The regression of work engagement on sleep quality showed that sleep quality was a positive and significant predictor of work engagement (β = 0.20, *p* < 0.05), yielding support for Hypothesis 1 (see Table 2), which stated that sleep quality should be positively related to work engagement.

**Table 2:** Results of regression analyses predicting work engagement.

	**Step 1**	**Step 2**
Intercept	2.61	0.75
Sleep quality	0.20^*^	−0.03
Mental health		0.48^***^
Physical health		0.02
Total *R^2^*	0.04	0.21^**^
Delta *R^2^*	0.04*	0.17^***^

* *p < 0.05*.

** *p < 0.01*.

*** *p < 0.001*.

*Betas are depicted. Regression analysis, method enter - Step 1 included sleep quality as a predictor of work engagement, step 2 added mental health and physical health*.

Hypothesis 2 stated a positive effect of sleep quality on mental health (2a) and physical health (2b). Results of regression analyses showed that sleep quality had a significant positive effect on both mental health (β = 0.46, *p* < 0.001) and physical health (β = 0.25, *p* < 0.01; see Table 3). These results supported Hypothesis 2.

**Table 3:** Results for direct and indirect effects.

	**Predictor**	***R^**2**^***	***B***	***SE***	**β**	**95 % confidence interval**	
						**LLCI**	**ULCI**
Mental health	Sleep quality	0.23	4.95	0.74	0.48^***^	3.50	6.40
Work engagement	Sleep quality (direct)	0.21	−0.03	0.11	−0.03	−0.24	0.17
	Sleep quality *via* mental health (indirect)		0.29	0.07	0.23*	0.13	0.34
	Sleep quality (total)	0.04	0.25	0.10	0.20^*^	0.06	0.45
Physical health	Sleep quality	0.06	1.82	0.58	0.25^**^	0.67	2.97
Work engagement	Sleep quality (direct)	0.04	0.26	0.10	0.21^*^	0.06	0.47
	Sleep quality *via* physical health (indirect)		−0.01	0.03	−0.01	−0.07	0.06
	Sleep quality (total)	0.04	0.25	0.10	0.20^*^	0.04	0.36

* *p < 0.05*.

** *p < 0.01*.

*** *p < 0.001*.

*β, standardized coefficient; LLCI, lower limit confidence interval; ULCI, upper limit confidence interval*.

Hypothesis 3 stated that (a) mental health and (b) physical health should be positively related to work engagement. Results are depicted in Table 2 and showed a significant positive effect of mental health (β = 0.48, *p* < 0.001) and no significant effect of physical health on work engagement (β = 0.02, *p* = 0.783). When tested separately as a single predictor, physical health was still not a significant predictor of work engagement. Thus, Hypothesis 3a was supported, while Hypothesis 3b needed to be rejected. Mental health explained a significant amount of variance in work engagement (*R*^2^ = 0.21, *p* < 0.001).

Hypothesis 4 stated indirect effects of sleep quality on work engagement *via* mental and physical health. Table 3 shows the results of indirect effect models for Hypothesis 4. The indirect effect of sleep quality on work engagement *via* mental health was significant (β = 0.23, LLCI = 0.13, ULCI = 0.34), i.e., mental health was a mediator in the relationship between sleep quality and work engagement, confirming Hypothesis 4a. The direct effect of sleep quality on work engagement was not significant when mental health was included in the regression model (β = −0.03, LLCI = −0.24, ULCI = 0.17). Furthermore, it was not significantly different from zero, so we could assume that the present mediation was a full one. The indirect effect of sleep quality on work engagement *via* physical health was not significant (β = −0.01, LLCI = −0.07, ULCI = 0.06), i.e., physical health was not a mediator in the relationship between sleep quality and work engagement, so Hypothesis 4b needed to be rejected. The model consisting of sleep quality as a predictor, mental and physical health as mediators, and work engagement as the criterion explained 21% of data variance in the mediation model.

Hypothesis 5 stated overtime hours to moderate the relationship between (a) mental health and (b) physical health and work engagement. Results are depicted in Table 4. The interaction term between mental health and overtime was not significant in predicting work engagement (Mental Health × Overtime: Estimate = −0.02, *SE* = 0.12, *t* = −0.22, *p* = 0.829). We found the same result for the interaction term between physical health and overtime (Physical Health × Overtime: Estimate = −0.18, *SE* =0.18, *t* = −1.67, *p* = 0.097). Consequently, we had to reject Hypothesis 5.

**Table 4:** Results of moderation analyses predicting work engagement.

	**Work engagement**
	**Step 1**	**Step 2**
Intercept	3.48	3.49
Mental health	0.45^***^	0.45^***^
Overtime	0.19^*^	0.20^*^
Mental health × Overtime		−0.02
Total *R^2^*	0.25	0.25^**^
Delta *R^2^*	0.25	0.00
Intercept	3.48	3.52
Physical health	0.00	−0.05
Overtime	0.22^**^	0.35^**^
Physical health × Overtime		−0.18
Total *R^2^*	0.05	0.07
Delta *R^2^*	0.05^*^	0.02

* *p < 0.05*.

** *p < 0.01*.

*** *p < 0.001*.

*Betas are depicted*.

### Robustness Checks and Additional Analyses

Following recommendations for correlational and cross-sectional studies (58–60), we repeated all analyses controlling for age, gender, leadership position, and regular and actual weekly working hours. More specifically, we included these variables in the regression analyses testing Hypotheses 1 – 3 as main effects (see Supplementary Table 1). Taking into account employees' age, gender, leadership position, and regular and actual weekly working hours as control variables did not affect our results regarding acceptance or rejection of hypotheses.

Moreover, we also tested gender, leadership position, age, and weekly working hours as moderator(s) of the relationships between sleep, mental health, and work engagement. That is, we investigated if certain relationships were different for, e.g., men vs. women or if certain relationships only existed for a certain group of participants, e.g., only for older participants. Interaction analyses revealed no significant interaction effects between the control variables and any of the predictor variables on any of the outcome variables. This means that the results equally apply to both men and women, employees in leadership positions and in non-leadership positions, older and younger employees, as well as employees working more or less hours. However, leadership position was significantly related to overtime hours and work engagement and that including leadership position as a covariate in the regression analyses erased the effect of overtime hours on work engagement.

Because mental health increases and physical health decreases with age, it is important to consider age-specific norms when interpreting scores on the SF-12 physical health and mental health subscales (61). Thus, we repeated all analyses once more, using the age-corrected difference scores for mental health and physical health (see Supplementary Table 2). Difference scores were calculated comparing each participant's index to the mean index for their age group. The analyses in which we used the age-corrected difference scores did not change the interpretation of the findings.

## Discussion

### Summary and Discussion of Results

The results largely support our conceptual model and highlight the relevance of sleep and health for employees' work engagement. In line with our predictions derived from the JD-R framework and the Effort-Recovery model, employees' sleep quality was indirectly and positively related to their work engagement *via* their mental health. The results of the current study thus suggest that (a) mental health does serve as a personal resource and that (b) both mental health and sleep quality can trigger motivational processes in line with the JD-R model. This highlights how valuable good sleep and mental health are for indicators of interest in the context of work, such as work engagement, a desirable experience for employees. Since work engagement is closely related to work performance, sleep and mental health might also boost work performance (15). Furthermore, the results allow a broader perspective on the JD-R model, since they provide a first hint of mental health's role as a personal, non-work resource in the context of the model, which has not received attention thus far.

Contrary to expectations, physical health did not serve as a link between sleep quality and work engagement. While sleep quality and physical health were significantly connected, physical health and work engagement were not. This result suggests that physical health does not serve as a relevant personal resource in the current study.

A possible explanation for why mental health turned out to serve as a personal resource in this context, while physical health did not, is that we investigated our model in a sample of white-collar workers. White-collar workers report higher psychological demands, whereas blue-collar workers report higher physical demands and more physical health complaints (62). Consequently, for white-collar workers, mental health should be more relevant for the affective-motivational state of work engagement (16, 63) than physical health. Equivalently, physical health should be more relevant for blue-collar workers. This assumption coincides with de Jonge's and Dormann's *Triple-Match Principle* (63) (see below for more details). In a different sample consisting of blue-collar workers, physical health might fit better into the JD-R model and our conceptual model. Thus, future studies might want to investigate physical health as a resource in a sample of blue-collar employees.

Based on the observation that our sample consists of white-collar workers arises another possible explanation for the fact that we could not find similar effects on work engagement for mental health and physical health. Since we excluded shift workers and blue-collar workers, our sample might be rather homogeneous regarding the physical health status. Low variance in our data regarding the physical health component of the SF-12 score could explain the lack of a connection between physical health and work engagement. The SF-12 is composed in a way that allows to compare individual scores to the norm mean index of 50.0 (*SD* = 10.0) (61). The mean index in our sample was *M* = 52.93, *SD* = 7.06, implying that participants were healthier than average, and their health status was relatively homogeneous. Therefore, we cannot rule out this argument as a possible explanation.

According to the JD-R model, overtime and mental health or physical health should have interacted in their prediction of work engagement due to their nature as personal resources and indicator for job demands, which they did not. Following the *Triple-Match Principle* (63), one possible reason for the rejection of this assumption is that health is not the appropriate resource to cope with overtime or job demands causing overtime. The Triple-Match Principle states that the more demands, resources and outcome variable match one another (triple match), the likelier there will be an interaction between demands and resources in predicting the outcome variable. In order to make a match, variables must be classified as belonging to the same category. For example, emotional demands and emotional resources likely predict emotional outcomes, whereas cognitive demands and cognitive resources likely predict cognitive outcomes. The likelihood for a joint prediction will decrease for a double, single or no match. If, in this case, there is no triple or double match between overtime, health, and work engagement, this could possibly explain why there was no interaction between overtime and health in predicting work engagement. Another explanation could be that physical health operates as a hygiene criterion for work engagement (e.g., like safety at work does for job satisfaction) (64): Physical health's absence could cause work engagement to decline, but its presence is not a booster for work engagement in the face of a job demand or its indicator, i.e., overtime. Finally, one more explanation might be that overtime as an indicator for job demands simply does not work for the JD-R model in the same way as do job demands themselves.

It should be noted that mental health and physical health turned out to be unrelated to each other (*r* = 0.02, *p* > 0.05). This means that, in our sample, mentally healthy employees were not physically healthy as well, even though theories of psychosomatic links (10) and results of empirical research (65) suggest that mental and physical health do have a connection. This finding reveals a particularity of our sample.

Interestingly, overtime as an indicator for job demands was *positively* related to work engagement. This might be a hint at the nature of job demands represented by overtime. Time pressure and high workload, two job demands potentially causing employees to work overtime, have been identified as challenge job demands rather than hindrance job demands (66–68). Challenge demands and resources can have similar effects on work engagement (69). However, when conducting robustness analyses considering age, gender, and leadership position as control variables, the positive relationship between overtime and work engagement disappeared. Further investigations revealed that leadership position was the variable responsible for the effect to disappear, showing that employees in a leadership position report more overtime hours and also more work engagement than employees in non-leadership positions.

### Limitations

Our study was designed as a cross-sectional study. Future researchers conducting studies on this topic might want to choose a longitudinal diary design to be able to explore the links between day-to-day fluctuations in sleep and employee experiences and behavior. They might even consider long-term studies to test for long-term effects of sleep quality on changes in health status and changes in work engagement. This might add useful insights to the results from our “momentary snapshot” study.

Another limitation resulting from our study design concerns the monocausal direction of the investigated relationship between sleep quality, health, and work engagement. Sleep quality, health, and work engagement might as well be reciprocally related: Work engagement might depend on sleep quality and mental health and, at the same time, predict sleep quality and mental health. Indeed, previous research provides some support for this idea, showing that work engagement can foster sleep quality. People experiencing more work engagement have less unfinished tasks and lower rumination and subsequently sleep better (70).

Subjective sleep quality has not been sharply defined and correlations with subjective sleep quality indicators, like wake after sleep onset or total sleep time, are medium to low (52). Also, subjective and objective measures for sleep quality do not correlate strongly (52, 71). Therefore, we chose to assess sleep quality using a widely accepted method, which is a single item retrieved from the PSQI (50). This single item is endorsed by Krystal and Edinger (52) in their article about sleep assessments. Still, in future studies, objective measures such as wrist actimetry might be utilized to triangulate the assessment of sleep quality.

### Practical Implications

Our study's results reveal some practical implications in the sense of prevention measures aimed at both environmental and behavioral change to be implemented. First, the results suggest that sleep quality is crucial for an employee's mental and physical health and their affective-motivational state at work, represented by work engagement.

Therefore, to foster work engagement, sleep quality may be enhanced. There are several possibilities to achieve better sleep quality, e.g., practicing sleep hygiene strategies (72), like avoiding exposure to blue light before going to bed, i.e., not watching TV or using a smartphone, or strategies for better recovery after work (73). A strategy for better recovery after work is fostering recovery experiences, i.e., mastery of challenging goals, mental detachment from work, relaxation and experience of control in leisure time. Research has shown that employees engaging in sleep hygiene practices experience higher self-regulatory capacity and higher work engagement (17), supporting this recommendation. Organizations may offer talks and speeches on these and other health-related issues (e.g., coping with stress) in order to help employees to promote their sleep quality.

Caring for their own sleep quality offers an opportunity to foster employees' self-regulation that should be supported and trained. Enabling employees to actively and consciously improve their sleep leads to creating more productive employees (74). Therefore, human resource departments that address personnel development should offer trainings and workshops aiming at employees' consciousness and awareness for the topic and at their self-regulation skills. Still, apart from providing possibilities for employees to become active agents for their health, organizations and leaders need to ensure that job characteristics allow employees to get enough restorative sleep. For example, previous research has found demands like time pressure, effort-reward imbalance and unfinished tasks to significantly affect sleep quality negatively *via* rumination (75). High performance expectations by leaders aggravate this effect (39). Therefore, not only should employees be trained to practice sleep-promoting skills, but also should leaders' awareness for the effects of their actions be raised.

Second, another topic that employees should be aware of is taking care of their own mental health. Mentally healthy employees turned out to experience significantly more work engagement in this study, so enabling employees to improve their own mental health might be beneficial for organizations. Raising employees' awareness by offering relevant health-oriented programs or conveying concrete self-care skills *via* coaching are two possible strategies to foster employees' mental health. Corporate health management and corporate health promotion are important approaches to create a basis for offering such programs. This study also revealed that promoting sleep quality is one of the basic preconditions for a healthy mind. Further, health-oriented leadership and other employee-oriented leader behavior should be encouraged among supervisors (76) in order to create a healthy work atmosphere and healthy work conditions for employees. Previous research has shown how transformational leadership supports employees in pursuing a healthy lifestyle, e.g., by fostering their work-life-balance (77).

## Data Availability Statement

The original contributions presented in the study are included in the article/Supplementary Material, further inquiries can be directed to the corresponding author/s.

## Ethics Statement

Ethical review and approval was not required for the study on human participants in accordance with the local legislation and institutional requirements. Written informed consent for participation was not required for this study in accordance with the national legislation and the institutional requirements.

## Author Contributions

JK made contributions to the conception and design of the study. RS conceptualized the model, analyzed, interpreted the data, and drafted the manuscript. RS and JK revised the manuscript for important intellectual content. All authors contributed to the article and approved the submitted version.

## Conflict of Interest

The authors declare that the research was conducted in the absence of any commercial or financial relationships that could be construed as a potential conflict of interest.
