# Supplementary material for: Fueling Work Engagement: The Role of Sleep, Health, and Overtime
Source: Front Public Health. 2021 May 20;9:592850. doi: 10.3389/fpubh.2021.592850 (PMC8172578; doi:10.3389/fpubh.2021.592850)
Supplement: Supplementary file 1 [file Data_Sheet_1.docx]

Supplemental Table 1

*Results of Regression Analyses Predicting Work Engagement Including Control Variables*

|  | Step 1 | Step 2 | Step 3 |
| --- | --- | --- | --- |
| Intercept | 4.01 | 3.46 | 2.52 |
| Age | .05 | .02 | -.10 |
| Gender | -.08 | -.13 | -.09 |
| Leadership position | .07 | .07 | .08 |
| Regular working hours | -.11 | -.14 | -.16 |
| Actual working hours | .13 | .16 | .15 |
| Sleep quality |  | .24** | .02 |
| Mental health |  |  | .50*** |
| Physical health |  |  | -.02 |
| Total *R^2^* | .03 | .08** | .27*** |
| Delta *R^2^* | .03 | .05** | .19*** |

*Note:* ** p* < .05. ** *p* < .01. *** *p < .*001. Betas are depicted. Gender is coded 0 = female, 1 = male. Leadership position is coded 0 = no leadership position, 1 = leadership position. Regression analysis, method enter - Step 1 included control
variables as predictors, step 2 added sleep quality, step 3 added mental health and physical health.

Supplemental Table 2

*Results of Regression Analyses Predicting Work Engagement Including Control Variables
and Using Age-Corrected Difference Scores for Mental and Physical Health*

|  | Step 1 | Step 2 | Step 3 |
| --- | --- | --- | --- |
| Intercept | 4.01 | 3.46 | 5.17 |
| Age | .05 | .02 | -.05 |
| Gender | -.08 | -.13 | -.09 |
| Leadership position | .07 | .07 | .08 |
| Regular working hours | -.11 | -.14 | -.16 |
| Actual working hours | .13 | .16 | .15 |
| Sleep quality |  | .24** | .02 |
| Mental health (Age-corrected) |  |  | .49*** |
| Physical health (Age-corrected) |  |  | -.001 |
| Total *R^2^* | .03 | .08** | .27*** |
| Delta *R^2^* | .03 | .05** | .19*** |

*Note:* ** p* < .05. ** *p* < .01. *** *p < .*001. Betas are depicted. Gender is coded 0 = female, 1 = male.
Leadership position is coded 0 = no leadership position, 1 = leadership position. Regression analysis, method
enter - Step 1 included control variables as predictors, step 2 added sleep quality, step 3 added age-corrected
difference scores for mental health and physical health.
